# Supplementary material for: Learning curve and surgical outcome of robotic assisted colorectal surgery with ERAS program
Source: Sci Rep. 2022 Nov 29;12:20566. doi: 10.1038/s41598-022-24665-w (PMC9709162; doi:10.1038/s41598-022-24665-w)
Supplement: Supplementary file 1 — Supplementary Information 1. [file 41598_2022_24665_MOESM1_ESM.zip › LSC Raw data-Ñ[▒K/092/O_ñw╝╨░O▒Kñσ.pdf]

正式摘要(電子簽章：000000199577751)

請選擇...reporter

臺中榮民總醫院

Operation Note

[redacted]

- 手術日期：2020/09/24
- 手術醫師：VS 蔣鋒帆，CR 黃俋霖，CR 楊曙榮，R 陳瑨夫
- Anesthesia : GE
- Position : Lithotomy
- Aseptic agents : Soap

Diagnosis

- Pre-operative diagnosis : Malignant tumor over R-S junction
- Post-operative diagnosis : Ditto.

Surgical Indication

- For oncological resection

Operation

- Laparoscopic Anterior resection and anastomosis - malignant

---

Finding

- Operative findings:
  1. A 2x 3 cm annular tumor at R-S colon, Borrmann type III, cT3N0M0
  2. No other gross tumor nor polyps was found
  3. There was no palpable liver metastasis, no visible peritoneal seeding

  Operative methods:
  1. Laparoscopic low anterior resection
  2. Anastomosis: double stapling, EndoGIA 60-4.1mm & EEA 29mm
  3. Proximal cut end: 10cm, distal cut end: 4cm
  4. Vessel ligation: IMA above left colic artery

Procedure

- General endotracheal anesthesia was induced and then the patient was placed in modified Trendelenburg lithotomy position. The abdomen was prepped and draped in standard sterile fashion. A small vertical incision, about 1 cm, was made above the umbilicus and then a 12mm trochar was inserted into peritoneal cavity by open method. Pneumoperitoneum was established with CO_2 inflation. Laparoscopy was inserted and other two 12mm trochars were inserted over RLQ and lower midline of abdomen. Two 5mm trochars were inserted at RUQ and LLQ of abdomen. Mesosigmoid colon was hold by grasper and dissection was made to separate mesocolon from retroperitoneum and further dissection was done toward mesorectum. IMA was identified and the endoclips were applied. IMA was divided below left colic artery. IMV was also divided after endoclips applied. Then the sigmoid colon was well mobilized and the presacral space was opened. The areolar tissue of presacral space was dissected. Then the upper rectum was divided by EndoGIA after rectal irrigation. The laparoscope and trochars were removed. A small midline incision was made over low abdomen. The rectum was pulled out and the middle sigmoid colon was resected as proximal cut point after mesocolon was divided. The specimen was excised and the cap of EEA was inserted into the proximal colon. Then the colon was put back into the peritoneal cavity and pneumoperitoneum was established again. End-to-end anastomosis was performed by EEA 29mm. The midline wound was closed with No.1 Vicryl suture. The wound was irrigated and closed with 3-0 Polysorb and 4-0 Vickryl. Under stable condition, the patient was sent to POR
- Estimated blood loss: minimum

- 器械數量: 正確
- 紗布數量: 正確

Image

- PAINT
    - colon & rectum

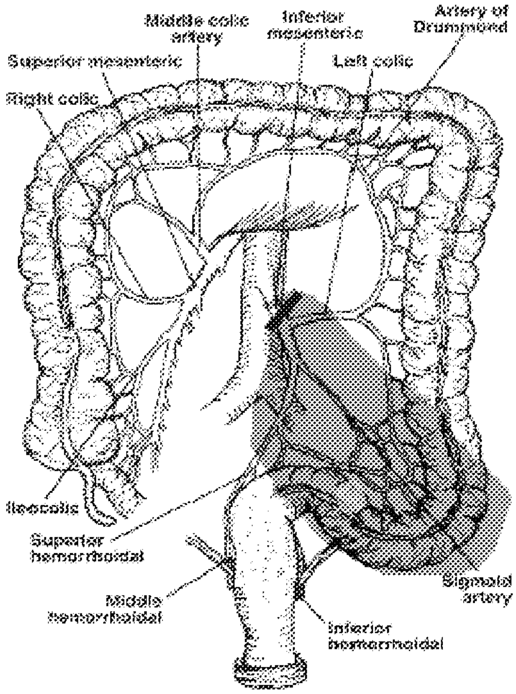

- 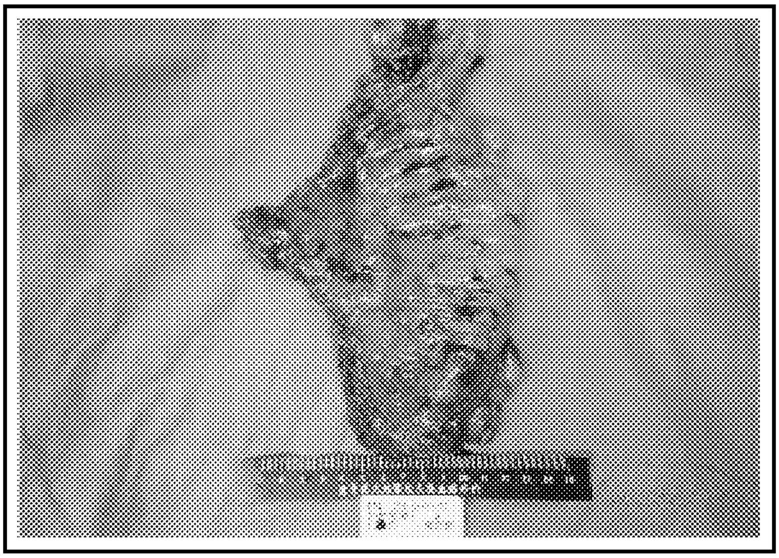

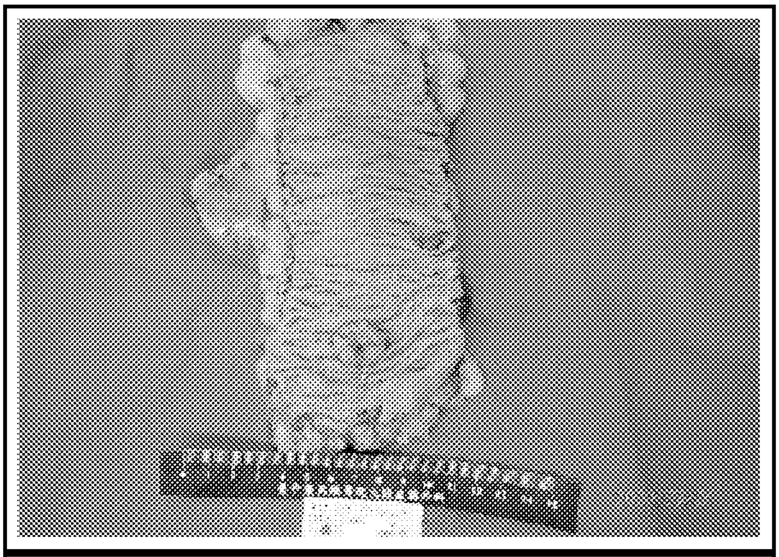

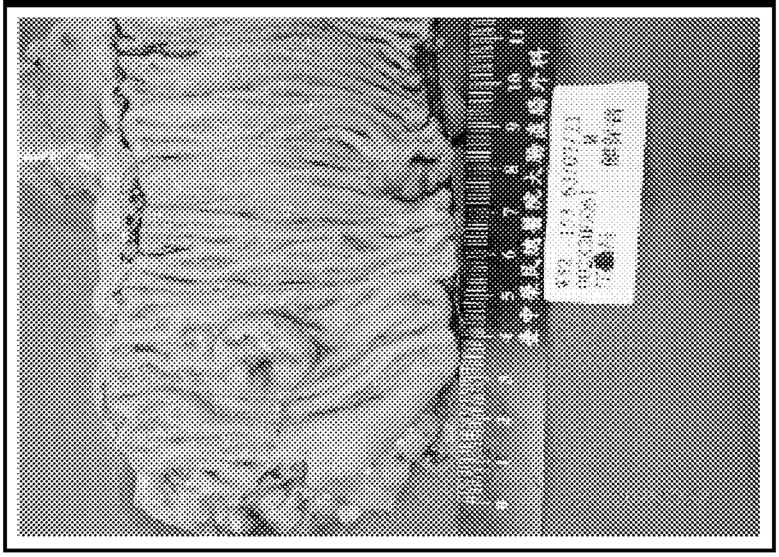

編輯主任 2062F蔣鋒帆、主治 2286H黃俋霖

正式摘要(電子簽章：000000199577751)

請選擇...reporter	

doctorRank:B
